# Molecular changes in intraocular fluid: implications for myopia

**DOI:** 10.7150/ijbs.101438

**Published:** 2024-09-30

**Authors:** Zewei Zhang, Lingfeng Lv, Dongmei Chen, Fang Li, Jibo Zhou

**Affiliations:** 1 Department of Ophthalmology, Shanghai Ninth People's Hospital, China.; 2 College of Health Science and Technology, Shanghai Jiao Tong University School of Medicine, China.; 3 Department of Laboratory Medicine, the First Affiliated Hospital, Fujian Medical University, China.

**Keywords:** Intraocular fluid, Aqueous humor, Vitreous, Myopia, Molecular changes

## Abstract

Myopia is the most common eye disease in the world which is caused by a mismatch between the optical power of the eye and its excessive axial length. Scleral remodeling, oxidative stress, inflammation, pathological states of angiogenesis and fibrosis and metabolism are closely associated with the onset and progression of myopia and the pathological changes that may ultimately result. Intraocular fluid is a collective term for the fluid within the eye, and changes in its composition can reflect the physiological and pathological status within the eye, with aqueous humor and vitreous being the commonly tested specimens. Recent studies have revealed potential changes in a variety of molecules in intraocular fluid during myopia progression. Abnormal expression of these molecules may reflect different stages of myopia and provide new perspectives for disease monitoring and treatment. Therefore, in this review, we systematically review the molecular changes in intraocular fluid associated with myopia, as well as the possible mechanisms, with a view to informing basic myopia research and clinical work.

## Introduction

Myopia represents a prevalent ocular disorder characterized by AL elongation 1, with its incidence escalating globally 2. As of 2010, the worldwide prevalence was recorded at 28.3%, with projections suggesting an increase to 39.9% by 2030 and 49.8% by 2050 2. This persistent rise underscores the imperative for a robust public health intervention. Myopia markedly impairs the educational achievements, quality of life, and employment opportunities of adolescents, imposing substantial burdens on individuals, families, and socioeconomic structures. Significantly, the economic impact attributed to uncorrected myopia was estimated at approximately US$244 billion globally in 2015, with East Asia experiencing the most significant economic strain 3. Moreover, myopia, whether low, moderate or high, is associated with a higher risk of several severe ophthalmic complications including cataracts, glaucoma, myopic macular degeneration, retinal detachment and even blindness 4. Therefore, in-depth understanding of the pathogenesis of myopia and searching for effective preventive and therapeutic methods have become important topics in ophthalmic research.

**Figure 1:**
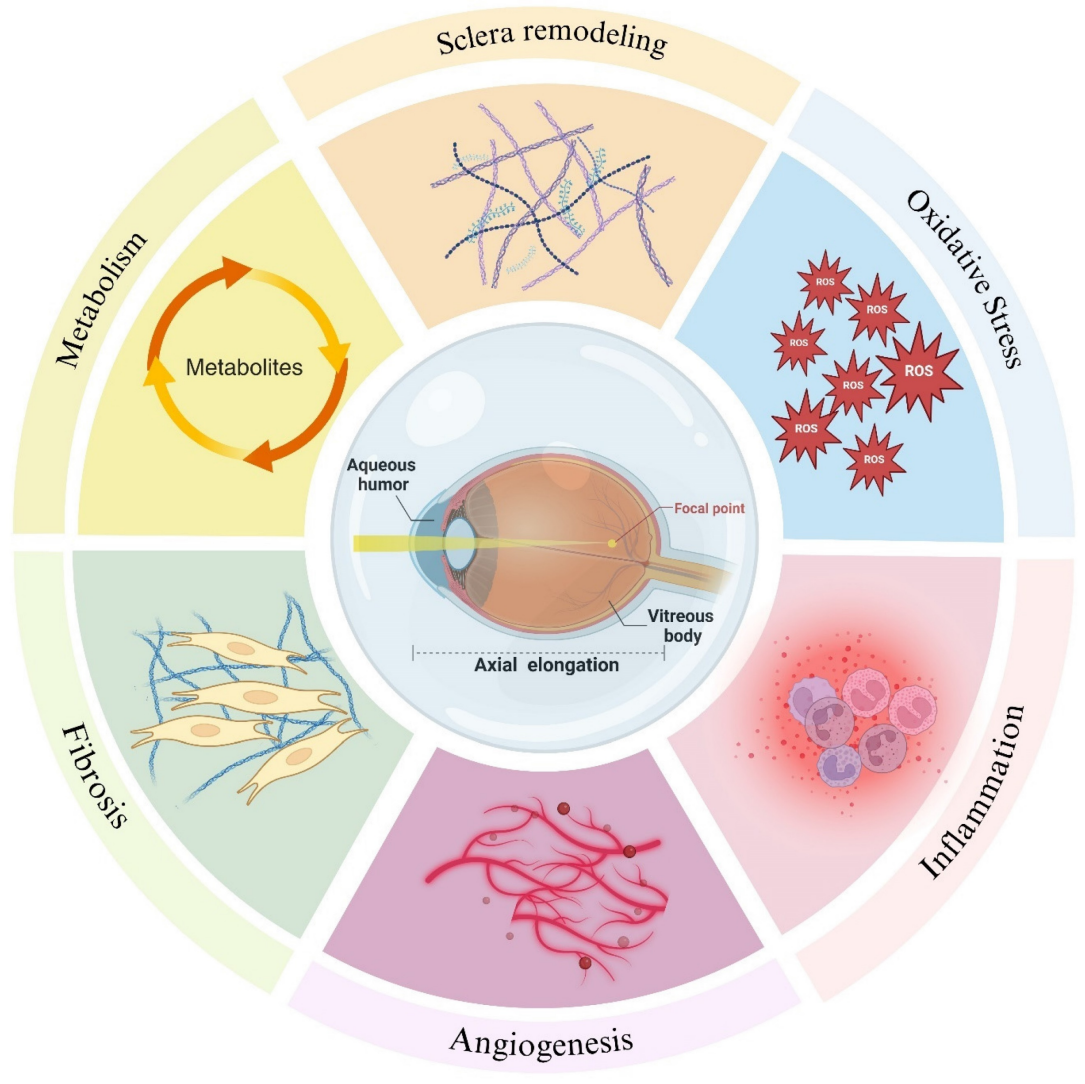
Molecular changes in intraocular fluid during the development of myopia reflect scleral remodeling, oxidative stress, inflammation, pathological states of angiogenesis and fibrosis and metabolism. Created with Biorender.com.

The intraocular fluid, encompassing AH and vitreous body, are integral in sustaining the ocular microenvironment. They provide essential nutrients to intraocular structures, regulate intraocular pressure, and facilitate the removal of metabolic byproducts 5,6. Extensive research substantiates that a diverse array of biomolecules within intraocular fluid is intricately linked to myopia progression through various biological processes such as scleral remodeling, oxidative stress, inflammation, the pathological states of angiogenesis and fibrosis and metabolism (Figure 1). Fluctuations in the molecular composition of intraocular fluid may signify critical phases in the development of myopia (Table 1). This review endeavors to elucidate the correlations between molecular alterations in intraocular fluid and myopia progression, aiming to furnish novel insights and directions for future investigations.

## Scleral remodeling

The sclera is instrumental in the initiation and progression of myopia, with alterations in its biomechanical properties, particularly the imbalance between degradation and synthesis of the scleral ECM, forming the structural foundation for AL hyperextension 7. As a microenvironment interacting directly with the sclera, shifts in the molecular composition of intraocular fluid can reflect the state of scleral remodeling to some extent. Subsequent research has revealed that variations in the expression of MMPs, TIMPs, and TGF-βs within the intraocular fluid are intricately linked to the process of scleral remodeling.

MMPs, a family of zinc-dependent endopeptidases involved in the degradation of a variety of proteins in the ECM, such as collagen and elastin, while TIMPs serve as specific inhibitors of MMPs 8. Thus, the balance between MMPs and TIMPs is critical for scleral remodeling 7. Animal model studies on myopia have demonstrated that increased expression of MMP-2 correlates with reorganization of the scleral structure and ECM remodeling 9,10. Furthermore, enhanced MMP-2 activity and reduced TIMP-2 activity have been observed to contribute to scleral remodeling in guinea pig 11, mouse 12 and chick 13,14 models. Theoretically, TIMPs, as inhibitors of MMPs, should be reduced in myopic progression, but some studies shown that TIMPs expression decreases with increasing MMP-2 15,16, which may be related to the *in vivo* homeostasis hypothesis 17,18. Interestingly, a recent study have shown that low doses of TIMP-2 promote MMP-2 activation, whereas high doses inhibit its activation, reduce collagen degradation, and shorten the AL, suggesting that TIMP-2 supplementation may be helpful in controlling myopia progression 19. These findings indicate a nuanced regulatory interplay between MMPs and TIMPs in the remodeling of the sclera.

Molecular changes associated with myopia have also been identified in human intraocular fluid as well. In an observational study involving patients with HM or cataract, intraoperatively removed AH was examined and found to contain MMP-2, MMP-3, TIMP-1, TIMP-2, and TIMP-3, but MMP-1 was not detected. Statistical analysis revealed that the levels of MMP-2, TIMP-1, TIMP-2, and TIMP-3 were positively correlated with AL, whereas MMP-3 showed no correlation 20. Considering the interaction between MMPs and TIMPs, further analysis showed that MMP-2 levels were elevated in AH in patients with HM, while MMP-3 levels remained unchanged. TIMP-1, TIMP-2, and TIMP-3 levels were positively associated with MMP-2 levels, but there was no significant association between MMP-3 and the levels of TIMP-2 or TIMP-3 and the TIMP- 1 levels were only slightly correlated with MMP-3 levels 21. These results align with the hypothesis that an increase in TIMPs is a compensatory response to maintain homeostasis. Additionally, changes in the vitreous body associated with myopia have drawn research interest. Zhuang *et al.* obtained vitreous samples from highly myopic patients undergoing vitrectomy, finding significantly elevated MMP-2 activity and levels, as well as an increased MMP-2/TIMP-2 ratio compared to controls 22. In myopic macular degeneration, MMP-2 levels were significantly elevated and positively correlated with AL 23.

There are three major TGF-β isoforms in mammals, which are crucial in regulating cell growth, differentiation, immune responses, and ECM formation 24. TGF-βs have a strong association with myopia. For instance, TGF-β2 levels are significantly elevated in both the retina and sclera of chicks with FDM 25. In tree shrews with experimentally induced myopia, a decline in various TGF-β isoforms correlates with AL elongation 26. In human studies, Zhu *et al.* analyzed AH sample from post-cataract surgery using real-time polymerase chain reaction and immunofluorescence staining, and found that TGF-β2 expression in the AH of patients with HM exceeded that of controls 27. Consistent results were also found in a recent study, which demonstrated that TGF-β2 levels in AH increased by 3.43% for each 1 mm increase in AL 28. Moreover, a prospective study revealed significantly higher TGF-β2 levels in the AH of patients with highly myopic cataracts compared to those with non-highly myopic cataracts. While TGF-β1 levels were not significantly altered, they were found to correlate with the patient's age and AL 29. Notably, this study found that TGF-β2 levels in the second eye of patients with HM were higher than those in the first eye, which may be related to the fact that cataract surgery promoted TGF-β2 expression in the other eye. Although the majority of research underscores a significant role for TGF-β, particularly TGF-β2, in myopia development, some studies report no significant differences. Zhuang *et al.* used enzyme-linked immunosorbent assay to measure TGF-β2 concentrations in the vitreous of HM patients and found them comparable to controls 22. This disparity might be attributed to differences in the environments of the AH and vitreous or variations in control group selection. Given the importance of TGF-β in the development of myopia, a Meta-analysis explored the relationship between TGF-β gene polymorphisms and myopia, and found that the rs4803455 and rs1800469 loci of TGF-β1 were associated with myopia, suggesting that further research and treatment of myopia based on these findings may be important. Yet rs1982073 and rs2241716 for TGF-β1 and rs7550232 for TGF-β2 were not significantly associated with myopia 30, indicating that further studies are still needed to explain these inconsistent results.

In summary, altered biomechanical properties of the sclera, particularly an imbalance between the degradation and synthesis of the ECM, form the basis of myopic AL hyperextension. Changes in MMPs, TIMPs, and TGF-βs in intraocular fluid are closely associated with scleral remodeling, and these molecules may serve as potential indicators of myopia progression. Given the complexity of myopia mechanisms and the inconsistency of findings, more in-depth studies are still needed in the future.

## Oxidative Stress

Oxidative stress is a state of imbalance between the production of ROS in the intra- and extracellular environment and the antioxidant defense system 31. An excessive buildup of ROS in the intraocular fluid has been linked to the onset of various ophthalmic conditions, including cataracts, glaucoma, and retinal disorders 31. Studies have pointed to a significant association between oxidative stress and the development of myopia. At the retinal level, proteomic analysis of samples from rabbit models of myopia revealed that oxidative stress may be a key mechanism in the progression of myopia 32. Another investigation in a chicken model induced by FDM revealed that the expression of neuronal NO synthase was increased, and that antioxidant intervention could mitigate myopia progression 33. At the scleral level, intense ROS release during oxidative stress may induce apoptosis in scleral cells, thereby fostering the growth of myopic sclera 34. In patients with HM, the substantial reduction of antioxidant enzymes and other protective agents can lead to cellular damage, apoptosis, and potentially impair visual functions 35.

In recent studies, the relationship between oxidative stress levels in the intraocular fluid and myopia development has garnered attention. T-AOC, an indicator of all antioxidant substances, and total nitrite/nitrate levels, an important indicator for assessing the state of oxidative stress, were measured in the eyes of middle-aged and elderly HM patients with AH. It was found that T-AOC values were significantly lower and total nitrite/nitrate levels were significantly higher in patients with HM, reflecting higher levels of oxidative stress in the intraocular environment. Further analysis revealed that T-AOC was negatively correlated with refraction and AL, indicating that the lower the T-AOC value, the greater the degree of myopia. Whereas, total nitrite/nitrate levels were positively correlated with refraction and AL, indicating that higher levels of oxidative stress were associated with more myopia 36.

Similar results were obtained in our study, where T-AOC levels were significantly lower in patients with AL ≥ 28 mm compared to patients with AL ≤ 26 mm in the testing of AH samples collected from 40 young patients (70 eyes) undergoing implantable collamer lens surgery. Additional correlation analysis confirmed a negative association between T-AOC and AL 37. The above results were consistent in both young and elderly patients with HM, emphasizing the potential role of oxidative stress in the development of myopia. Regarding the vitreous, examination of samples from animal models of myopia combined with bioinformatics analysis suggests that oxidative stress and lipid metabolism are pathways involved in compensatory eye elongation 38.

Overall, oxidative stress appears to play a significant role in myopia development within the intraocular fluid environment, particularly in HM patients. The reduced antioxidant capabilities and increased oxidative markers highlight a pathogenic process that potentially accelerates myopia by altering the biological functions of the retina and sclera.

## Inflammation

Inflammation is the body's natural response to harmful stimuli and typically benefits the organism when controlled 39. It serves as a key factor in the development of myopia, as evidenced by studies indicating that the expression of ocular inflammatory cytokines escalates with the progression of myopia in various animal models including tree shrews 40, guinea pigs 41, hamsters 42,43, and chicks 44. These studies suggest that an increased state of ocular inflammation accelerates the development of myopia. Additionally, patients with inflammatory diseases have been reported to exhibit a higher prevalence of myopia 43,45. In comparison, various markers of inflammation such as elevated circulating neutrophils, reduced monocytes, eosinophils, and lymphocytes 46, along with increased neutrophil-to-lymphocyte ratios and platelet-to-lymphocyte ratios, demonstrate an imbalance in patients with high and pathological myopia. These findings suggest that individuals with myopia may experience a systemic inflammatory state^47^,^48^.

Intraocular fluid is one of the main sites of inflammatory response in myopia development. Research shows that myopic eyes exhibit elevated levels of inflammatory cytokines in the vitreous or AH compared to non-myopic eyes. For instance, significant increases in cytokines such as MCP-1, IL-5, IL-6, IFN-γ, IP-10, eotaxin, macrophage inflammatory protein-1α, IL-4, and granulocyte colony-stimulating factor have been documented in the vitreous of patients with HM 49. Similarly, increased levels of IL-6, IL-8, IL-10, IP-10, and MCP-1 have been observed in HM and its complications, further indicating a pro-inflammatory state in highly myopic eyes 50. In the AH of patients with highly myopic cataracts, the expression of IL-1ra was decreased, whereas the expression of MCP-1 was elevated, suggesting that the anterior chamber of the eye may be in a pro-inflammatory state 51. Moreover, the positive correlation of MCP-1 with AL reinforces its association with myopia progression 28. IL-6 and IL-8, as typical inflammatory factors, are similarly significantly altered in the atrium in addition to the vitreous. It has been found that IL-6 and IL-8 are significantly higher in highly myopic eyes than in non-highly myopic eyes and have a significant positive correlation with the AL 52,53. Notably, the tight junctions of the cornea are diminished in eyes suffering from allergic conjunctivitis, leading to increased levels of intraocular inflammatory cytokine expression, which may be related to cytokine penetration, with high levels of MCP-1, IL-6, IL-8, and TNF-α being recognized as a potential contributor to the promotion of retinal inflammation and myopic progression 54 , indicating a more profound effect of inflammation on myopic progression. The development of mCNV is closely associated with the upregulation of inflammatory cytokines. It has been shown that the inflammatory cytokines IL-6, IL-8, IL-10, IP-10 and MCP-1 were significantly increased in AH in the eyes of highly myopic patients with and without mCNV compared to controls 50. However, the evidence for an association between myopia and inflammation is not consistent. One study found no significant correlation between AL and concentrations of inflammatory cytokines such as IL-1β, IL-6, IL-8, IL-10, IL-12p, and TNF-α in AH of cataract patients with AL ranging from 22.6 to 31.5 mm 55. Similarly, our research detected no significant changes in TNF-α 37. Additionally, other investigations reported that IL-6 and IL-8 levels were not significantly elevated compared to controls 23,51, and that log-transformed concentrations of MCP-1, sICAM-1, and svCAM-1 showed no correlation with refractive error or AL 56. Such discrepancies could stem from variations in sample size, testing methodologies, control group selection, and inclusion criteria. In particular, most of the samples were from cataract patients, which can have an effect on the molecular changes of intraocular fluid, and this may be one of the reasons for the inconsistent results of the study. Therefore, younger patients should be included as controls in future studies. Our recent study took this aspect into account by including non-cataract patients aged 18-45 years and found that IL-1β and IL-6 concentrations were significantly higher in the AH of patients with AL ≥28 mm compared with those with AL ≤26 mm, with IL-6 positively correlating with AL 37, presenting a consistent performance with previous studies 52,53.

Given the role of inflammation in various ocular diseases and the complex mechanisms underlying myopia, further research is essential to clarify the relationship between inflammation and myopia and to uncover the underlying mechanisms.

## Angiogenesis

VEGF is a critical bioactive protein integral to angiogenesis 57. Studies have shown that aberrant VEGF expression is strongly associated with a variety of serious ocular diseases 57. The involvement of VEGF in myopia development is increasingly acknowledged. A study reported that intravitreal injection of VEGF165 effectively slowed myopia progression in guinea pig models of FDM-induced myopia 58. Significant changes in VEGF have also been found in the human eye. Most studies noted that in patients with HM and mCNV, VEGF levels in the AH were significantly decreased and showed a negative correlation with AL 23,36,52,53,55,56,59-62. Notably, there were variations in the experimental procedures of the researchers. The study by Sawada *et al.* excluded patients with uveitis, myopic atrophy, and diabetes mellitus to minimize confounding by VEGF expression, but did not establish a normal control group and was a cross-sectional study, which limited the inference of causality 61. Shin *et al.* enhanced the robustness of their findings through subgroup analysis but included patients with conditions that could compromise the generalizability of the results 62. Yuan *et al.* reported a decrease in AH VEGF levels corresponding to increases in AL, but did not account for the impact of intraocular volume on these levels 53. Conversely, Wong *et al.* controlled for AL in their analysis, yet faced limitations due to the small scale and cross-sectional nature of their study 23. In vitreous samples, Zhu *et al.* and Hu *et al.* found that VEGF concentrations decreased as AL increased 55,59. However, one study showed significantly higher levels of VEGF in the vitreous of patients with HM, and the authors hypothesized that this may be related to a low-grade inflammatory state in the eye rather than differences in the type of retinopathy 49.

In mCNV patients, the role of VEGF has also received attention from researchers. It was reported that VEGF levels in AH of mCNV patients were lower compared to controls, but there was no correlation between VEGF and AL. It is hypothesized that VEGF may be mainly localized in the retina and choroid, resulting in low levels in AH. Another theory proposes that due to the extended AL and consequently larger intraocular volume in HM patients, VEGF might be diluted in the anterior chamber and vitreous cavity 63. Notably, low VEGF levels in the AH of patients with mCNV have also been found to be accompanied by other cytokine imbalances^64^. Moreover, VEGF levels were notably higher in the vitreous of highly myopic patients with mCNV compared to controls, although no disparity was noted in AH levels. This may reflect altered hemodynamics in the dilated retinal pigment epithelium cells and choroid in the posterior pole 50. In response to the inconsistency of test results in AH as well as vitreous samples, the study introduced a novel approach by considering VEGF mass—calculating the total amount of VEGF (concentration multiplied by volume) rather than concentration alone, accounting for changes in AL and vitreous volume to more accurately assess VEGF's bioactivity within the eye 50.

PEDF is a potent inhibitor of angiogenesis 65. Typically, in normal ocular tissues, the regulation of angiogenesis is governed by the equilibrium between VEGF and PEDF 66. Despite expectations of elevated PEDF levels in highly myopic eyes to counterbalance reduced VEGF, it has observed diminished PEDF levels in the AH of patients with HM 67. Conversely, while one investigation found no significant disparity in PEDF levels between highly myopic individuals and control subjects, the VEGF/PEDF ratio was notably reduced in the highly myopic group 62. This suggests potential damage to the fundus and may partially clarify the predominant influence on VEGF secretion relative to PEDF in scenarios of fundus alterations triggered by HM, thus accounting for the unanticipated lower PEDF levels 23,62.

Angiogenesis is a prominent feature of PM, and anti-VEGF therapy has been widely used to inhibit this pathological process. Although measuring VEGF concentrations in intraocular fluid can shed light on angiogenic activity, it does not fully encapsulate the complex dynamics of angiogenesis. Emerging research suggests that evaluating the total VEGF content might offer a more holistic view of angiogenic processes. Moreover, maintaining the balance between VEGF and PEDF is crucial for the regulation of angiogenesis, making the VEGF/PEDF ratio a potentially superior metric for assessing the state of angiogenesis compared to the levels of individual factors.

## Changes in growth factors

Experimental animal studies have demonstrated that both topical and intraocular administration of multiple growth factors can influence externally induced AL elongation 68-70. These growth factors undergo specific changes in the intraocular fluid during the development of myopia. EGF is a biologically active polypeptide whose primary role is to promote cell growth, proliferation, and differentiation 71. One study identified a negative correlation between the total intraocular EGF and AL, indicating that EGF may be linked to pathological changes in the fundus in cases of HM 72. This was calculated by multiplying the EGF concentration by the intraocular volume in a method consistent with Zhang *et al.* 50. IGFs, crucial for tissue growth, cellular proliferation, and repair, are believed to play a significant role in myopia development 69,73. Insulin microarray analysis of AH and vitreous samples from patients with PM showed that insulin, IGF-2, IGF-2R, and IGFBPs were significantly increased in patients with PM 74. GDF-15, part of the TGF-β superfamily, participates in the TGF-β signaling pathway, influencing inflammatory responses and promoting fibrosis 75,76. HGF, extensively studied in various ocular diseases, including corneal and retinal disorders, has been shown to upregulate MMP-2 expression in scleral fibroblasts of myopic guinea pigs, potentially contributing to myopia development through the degradation of scleral ECM 70. PDGF was first isolated from platelet extracts in the early 1970s and classified as a mitogen for fibroblasts and mesenchymal-derived cells 77. Notably, levels of GDF-15, HGF, and PDGF-AA were found to be significantly higher in the AH of patients with HM compared to controls. Given the pro-fibrotic effects of these growth factors, researchers have hypothesized their involvement in the fibrotic processes associated with HM 36,60.

## Other potential molecules

**Figure 2:**
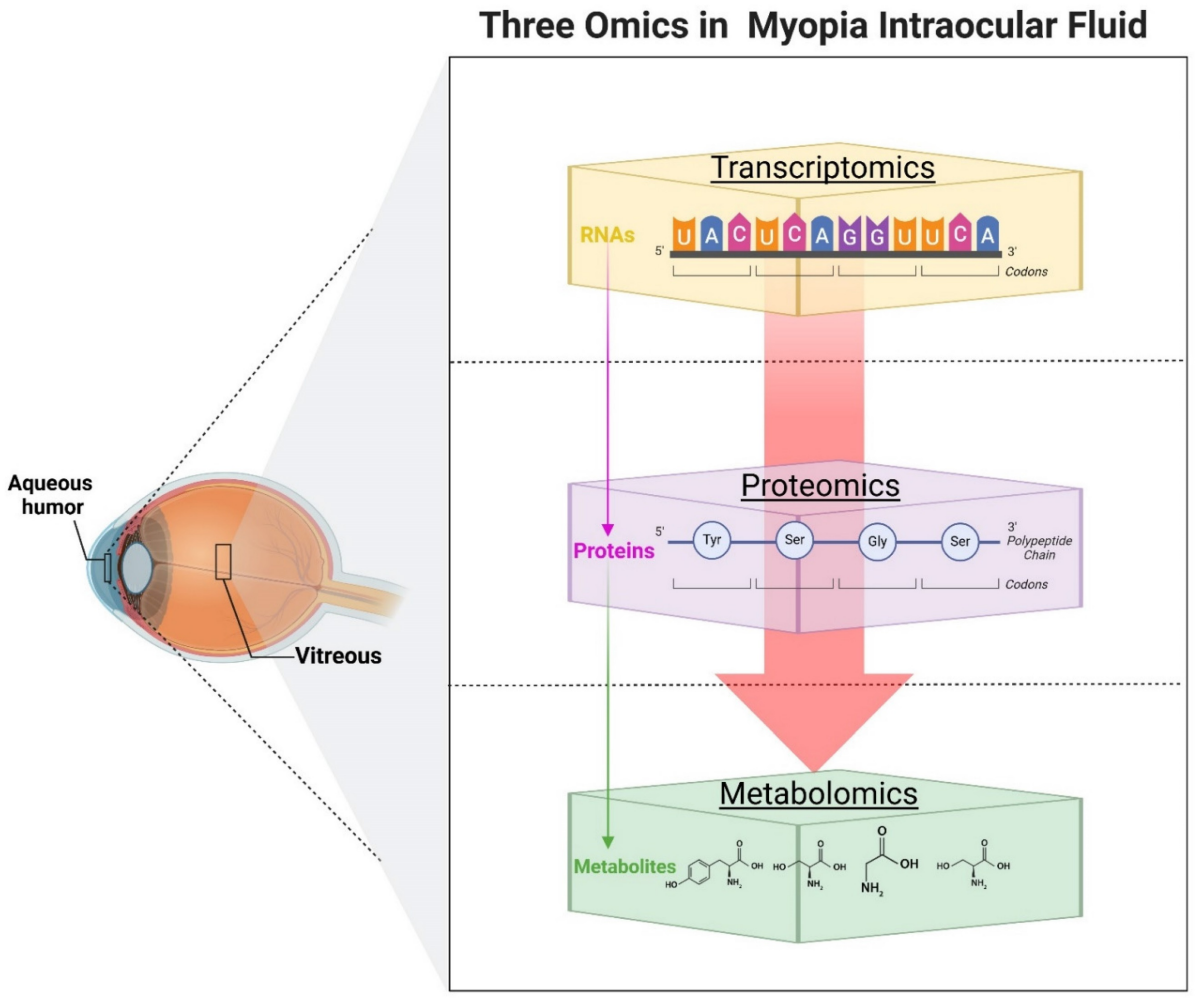
Overview of transcriptomics, proteomics, and metabolomics in analyzing molecular changes in intraocular fluid related to myopia. Created with Biorender.com.

With the advancement of omics technologies, researchers have obtained significant insights by analyzing AH or vitreous samples from myopic or highly myopic patients (Figure. 2). Transcriptomics and proteomics, two vital branches of omics research, have offered fresh perspectives in understanding the molecular underpinnings of myopia (Table 2). Transcriptomics have revealed an abundance of miRNAs in AH 78, often encapsulated within exosomes 79. The altered miRNA expression profiles are closely linked to myopia progression. Chen *et al.* first analyzed the miRNA expression profiles of exosomes in myopic patients' AH and found that the total RNA content in the myopic group was 2.78 times higher than that in the control group. By Open Array, they identified five myopia-specific miRNAs (has-miR-582-3p, has-miR-17-5p, has-miR-885-3p, has-miR-19b-3p, and has-miR-450b-5p), which may be six common myopia-related genes as potential targets 80. NGS was used to detect a total of 249 mature miRNAs and 17 novel miRNAs that were differentially expressed in myopia, which may be involved in the regulation of the TNF, MAPK, PI3K- AKT, and HIF-1 signaling pathways, and were verified by quantitative polymerase chain reaction 81. In another study, also using the NGS combined with immunofluorescence and western blot validation, it was concluded that hsa-miR-142-3p may be involved in the pathologic progression of HM by reducing collagen I in scleral fibroblasts by targeting TGF-β1 82. The researchers also noted whether there were corresponding changes in the vitreous of the patients. Detection of miRNAs in the vitreous using Low Density Arrays analysis revealed that let-7c was significantly upregulated in the vitreous of highly myopic eyes, while miR-200a was significantly downregulated. Further analysis showed that the up-regulated miRNA target genes involved MAPK and multiple inflammatory signaling pathways; the down-regulated miRNA target genes involved the PI3K/AKT pathway 83.

Proteomics serves as the one of the final steps downstream of the omics cascade, focusing on global protein measurement. An iTRAQ-based quantitative proteomics study identified 210 proteins differentially expressed in HM patients, with 123 proteins up-regulated and 87 proteins down-regulated in patients with HM 84. However, results from other ITRAQ-based studies varied, possibly due to differences in sample size and inclusion criteria. One found that 146 proteins were differentially expressed among proteins associated with the HM group compared with the control group, of which 97 proteins were upregulated and 49 proteins were downregulated in patients with HM 85. In another study, 58 differentially expressed proteins were identified, of which 32 were up-regulated and 26 were down-regulated. Bioinformatic analysis and confirmation by enzyme-linked immunosorbent assay suggested that PLG may be a candidate biomarker for HM. Its overexpression may lead to scleral thinning by affecting the composition of collagen fibers and ECM of the sclera 86. APOA1 has emerged as a significant protein in several studies, associated with myopia arrest in animal models 87 and identified in both chick embryo vitreous 38 and AH exosomes of HM patients 88. APOA1's role is supported by its implication in oxidative stress and lipid metabolism pathways 38. A proteomics study using old experimental methods indicated that DBP, albumin, and transthyretin were significantly increased in highly myopic eyes. The researchers suggested that DBP may be involved in the APOA1 arrest pathway and thus play a specific role in the development of myopia 89. The results of a study on PM also ultimately point to APOA1 90. Additionally, proteomics has extended to studying mCNV, with elevated levels of glial fibrillary acidic protein and complement-related proteins identified, primarily involving the JAK-STAT pathway 91.

Metabolomics, as another step in the final stage o in the cascade of omics, is increasingly elucidating the association between metabolite alterations in intraocular fluid and the progression of myopia (Table 3). Both AH and vitreous have been central to myopia-related metabolomic studies. Through untargeted approaches using CE-MS and LC-MS, over 40 metabolites have been identified. Higher levels of aminooctanoic acid, arginine, citrulline and sphinganine were observed in patients with HM compared to those with low myopia. These metabolites play pivotal roles in NO synthesis and cellular signaling, suggesting their involvement in myopia development by influencing intraocular pressure regulation and ocular growth. Additionally, low myopia patients exhibited higher levels of aminoundecanoic acid, dihydro-retinoic acid and cysteinylglycine disulfide. These metabolites may be linked to retinal health and antioxidant defense related to retinoid signal transduction, indicating a potential protective role at different myopia stages 92. Using GC-TOF-MS in an untargeted manner, 29 metabolites showed significant changes between HM patients and controls, with 27 increasing and 2 decreasing. Further metabolic network analysis highlighted the prominence of amino acid metabolism pathways. Specific amino acid metabolites, such as glutamine, N-alpha-acetyl-L-ornithine, nicotinoylglycine, and o-hydroxyhippuric acid, were markedly upregulated. Levels of oxalic acid, linoleic acid methyl ester, and thymidine also significantly increased, with the latter two being associated with enhanced ROS generation and oxidative stress in HM patients. The rise in oxalic acid might relate to calcium binding and subsequent impacts on calcium concentration, contributing to myopia progression. Thymidine levels are thought to correlate strongly with certain features of AL elongation 93. Shao *et al.* utilized LC-MS to analyze AH metabolites in patients with AL less than 24 mm (Group A), between 24-26 mm (Group B), and greater than 26 mm (Group C). Comparative and enrichment analyses across these groups revealed that differential metabolites were involved in Taurine and hypotaurine metabolism, vitamin B6 metabolism, pantothenate, and coenzyme A biosynthesis.

Further trend analysis and determination of differential metabolites based on FC values, coupled with receiver operating characteristic curve analysis and correlation analysis, identified 5-methoxytryptophol and cerulenin as potential biomarkers significantly associated with abnormal axial myopia 94. A study on PM, using an untargeted UHPLC-MS/MS approach, analyzed AH and vitreous humor samples from PM and non-myopic patients. The analysis revealed significant differences in 104 AH metabolites and 114 VH metabolites between the groups. Enrichment analysis identified four metabolomic pathways associated with PM: bile secretion, insulin secretion, thyroid hormone synthesis, and the cGMP-PKG signaling pathway. Additionally, ten amino acids were significantly associated with AL 74. Another study employing similar techniques investigated the metabolomic characteristics of vitreous in PM patients, identifying significant changes in 19 metabolites within the tryptophan metabolism pathway, notably elevated levels of L-kynurenine, indicative of increased oxidative stress and inflammatory response. Furthermore, significantly elevated uric acid levels were correlated with PM complications, suggesting its potential as a PM biomarker. The study also observed changes in amino acid (gamma-glutamyltyrosine and 4-guanidinobutyric acid) and lipid metabolites (L-carnitine, acetyl-L-carnitine and propionylcarnitine), associated with heightened oxidative stress in PM 95.

It is worth noting that multi-omics research has been gradually utilized in the exploration of disease mechanisms. As a multifactorial disease, the combined application of multi-omics can provide a more comprehensive and in-depth understanding of the changes in the development of myopia, especially in the screening of certain meaningful biomarkers. In conclusion, the application of omics technologies to AH and vitreous samples has uncovered crucial molecular alterations and potential biomarkers. These findings are instrumental in deepening our understanding of myopia's mechanisms, aiding in its diagnosis, and fostering the development of targeted treatment strategies.

## Conclusion

The etiology of myopia is shaped by an intricate interplay of genetic predispositions and environmental exposures, thereby complicating the elucidation of its underlying mechanisms. Despite considerable advancements in the field, a definitive account of the factors driving myopia progression remains elusive. Current findings on myopia are mostly based on animal and cellular experiments due to the difficulty of obtaining specimens from the human eye. With the popularization of cataract surgery and advances in vitrectomy surgery, we are able to obtain intraocular fluid, including AH and vitreous, in an ethical manner. However, the widespread adoption of cataract extraction and the refinements in vitrectomy techniques have facilitated the ethical collection of intraocular fluid, including AH and vitreous body, thus enriching our understanding of myopia at the molecular level. According to existing studies, there are corresponding changes in the molecules in the intraocular fluid during the progression of myopia as well as in the later stages of the pathology, which point to scleral remodeling, oxidative stress, inflammation, pathological states of angiogenesis and fibrosis and metabolism. Yet researchers face certain challenges in interpreting these changes. On the one hand, this may be due to the fact that the source of AH and vitreous molecules is not limited to the eye, but can partly come from plasma 96,97. On the other hand, limitations of the studies themselves, such as insufficient sample sizes, varying inclusion criteria, differences in statistical methods, and failure to adequately take into account the dilutional effect of AL elongation, may lead to inconsistent results. Moreover, the majority of human ocular specimens are derived from elderly patients undergoing cataract surgery, which may not offer comprehensive insights comparable to those obtained from younger individuals. Fortunately, researchers are gradually overcoming these challenges with improvements in study design and advances in assay technology. Developments in microscopic specimens and high-throughput assay technologies have led to more precise analysis of molecular changes in intraocular fluid. Researchers are increasingly recognizing the importance of robust study designs and have begun to consider the effect of AL on results, expand sample sizes, and include low myopia and younger patients as controls to improve the scientific validity of their studies. In summary, the study of molecular changes in intraocular fluid has been instrumental in shedding light on the mechanisms of myopia. Despite existing challenges, ongoing technological advancements and methodological improvements provide optimism for a more thorough and direct comprehension of myopia at the human level in the foreseeable future. Such advancements are poised to enhance the strategies for prevention and management of myopia.

## Figures and Tables

**Table 1:** Myopia-related molecular changes in human intraocular fluid

Molecules	Sample source	Molecular changes	Ref.1	AL association	Ref.2
MMP-2	AH, Vitreous	Increased	20-23	Positive correlation	20,23
TIMP-1	AH	Increased	20,21	Positive correlation	20
TIMP-2	AH, Vitreous	Increased	20-22	Positive correlation	20
TIMP-3	AH	Increased	20,21	Positive correlation	20
TGF-β1	AH	No Significant Change	29	Positive correlation	29
TGF-β2	Vitreous	No Significant Change	22	——	
	AH	Increased	27,29	Positive correlation	28
T-AOC	AH	Decreased	36,37	Negative correlation	36,37
Total nitrate	AH	Increased	36	Positive correlation	36
MCP-1	AH, Vitreous	Increased	28,49-51	Positive correlation	28
		——		No correlation	56
sICAM-1	AH	Increased	28	Positive correlation	28
	AH	——		No correlation	56
svCAM-1	AH	——		No correlation	56
IL-1β	AH	Increased	37	No correlation	37,55
IL-1ra	AH	Decreased	51	——	
IL-4	Vitreous humor	Increased	49	——	
IL-5	Vitreous humor	Increased	49	——	
IL-6	AH, Vitreous	Increased	37,49,50,52,53	Positive correlation	37,52,53
	AH	No Significant Change	23,51	No correlation	55
IL-8	AH	No Significant Change	23,51	No correlation	55
	AH, Vitreous	Increased	50,52	Positive correlation	52
IL-10	AH, Vitreous	Increased	50	No correlation	55
IL-12p	AH	——		No correlation	55
TNF-α	AH	No Significant Change	37	No correlation	37,55
IP-10	Vitreous	Increased	49,50	——	
IFN-γ	Vitreous	Increased	49	——	
Eotaxin	Vitreous	Increased	49	——	
MIP-1α	Vitreous	Increased	49	——	
G-CSF	Vitreous	Increased	49	——	
VEGF	AH, Vitreous	Decreased	23,36,52,53,62-64	Negative correlation	23,36,52,53,55,56,59,61-63
	Vitreous	Increased	49,50	Positive correlation	50
PEDF	AH	No Significant Change	23,62	No correlation	23,62
	AH	Decreased	67	——	
VEGF/PEDF	AH	Decreased	62	——	
EGF	AH	Increased	72	Positive correlation	72
IGF-2IGF-2RIGFBP-1IGFBP-2IGFBP-3IGFBP-4IGFBP-6	Vitreous	Increased	74	——	
GDF-15	AH	Increased	60	Positive correlation	60
HGF	AH	Increased	36,60	Positive correlation	60
PDGF-AA	AH	Increased	60	Positive correlation	60

**Table 2:** Transcriptomics and proteomics applied to the detection of myopia-related intraocular fluid molecules

	Sample source	Detection methods	Changes	Relevant mechanisms involved	Specific miRNA or protein	Ref.
miRNAs	AH	Open Array	The amount of AH RNA was up 2.78-fold in the highly myopic group	——	Has-miR-582-3p, has-miR-17-5p, has-miR-885-3p, has-miR-19b-3p and has-miR-450b-5p	80
miRNAs	AH	NGS	249 miRNAs and 17 novel miRNAs differentially expressed in myopia	TNF, MAPK, PI3K-Akt and HIF-1 signaling pathways	Has-let-7i-5p, has-miR-127-3p and has-miR-98-5p	81
miRNA	AH	NGS	miR-142-3p was upregulated and positively correlated with ocular AL	Targeting TGF-β1 to reduce collagen I in scleral fibroblasts	miR-142-3p	82
miRNAs	Vitreous	Low Density Array	Let-7c miRNA was upregulated and miR-200a was downregulated	Up-regulated: 23 pathways including MAPK pathways; down-regulated: 32 pathways including PI3K/AKT	Let-7c, miR-200a	83
Proteins	AH	iTRAQ	123 were up-regulated and 87 were down-regulated proteins	——	——	84
Proteins	AH	iTRAQ	97 were up-regulated and 49 were down-regulated	——	——	85
Proteins	AH	iTRAQ	32 up-regulated and 26 down-regulated proteins	PLG affects the composition of collagen fibers and ECM leading to scleral thinning	PLG	86
Proteins	AH	Conventional Method	Albumin; transthyretin, DBP were significantly elevated.	DBP may be involved in the APOA1 arrest pathway	Albumin, transthyretin, DBP	89
Proteins	AH	Label-free	63 up-regulated and 38 down-regulated	APOA1 as a stop signal for myopia progression may be a key protein and therapeutic target for PM	APOA1	90
Proteins	AH	Label-free	Glial fibrillary acidic proteins and complement-related proteins were elevated	JAK-STAT signaling pathway	Glial fibrillary acidic proteins and complement-related proteins	91

**Table 3:** Metabolomics of intraocular fluid components

Detection methods	Sample source	Altered biomarkers	Potential mechanisms	AL association	Ref.
CE-MS and LC-MS	AH	Aminooctanoic acid, L-arginine, citrulline, aminoundecanoic acid, L-cysteinylglycine disulfide trihydroxyphenyl-gamma-valerolactone, dihydropteroic acid, dodecanedioic acid, aminocyclohexanecarboxylic acid, butyryl-L-carnitine, pantothenic acid, didehydro-retinoic acid, sphinganine, histidinyl-phenylalanine, dimethylnonanoyl carnitine, PC(O-32:2)//PC(P-32:1), PC (42:6), C24 sulfatide, PC(P-42:2)//PC(O-42:3), laccer(d40:0), trihexosylceramide (d36:2), neuacagalcer(d42:2)	Oxidative stress, NO synthesis and retinoic acid signaling	——	92
GC-TOF-MS	AH	N-alpha-acetyl-L-ornithine, nicotinoylglycine, oxalacetic acid, o-hydroxyhippuric acid, oxalic acid, ribose, cis-gondoic acid, linoleic acid methyl ester, thymidine, phosphate, indole-3-acetamide, 2-aminophenol, 2-ketoadipate, 3-phenyllactic acid, cis-phytol, conduritol b epoxide, salicin	Amino acid metabolic pathways, oxidative stress and calcium ion binding	Thymidine	93
UHPLC-MS/MS	AH	SQH, inosine, uridine, hypoxanthine, pseudouridine, 3-nitro-L-tyrosine, phosphopyruvic acid, L-threo-3-phenylserine, DL-0-tyrosine, APK	Bile secretion, insulin secretion, thyroid hormone synthesis and cGMP-PKG signaling pathway	Serine, methionine, proline, creatine, lysine, arginine, tyrosine, threonine, glutamine, asparagine	74
	Vitreous	D-(-)-glutamine, hypoxanthine, prostaglandin G2, decanoylcarnitine, Dl-glutamic acid, L-threonic acid, L-(+)-citrulline, citramalate, L-pyroglutamic acid, KPH	Bile secretion, thyroid hormone synthesis, insulin secretion and cGMP-PKG signaling pathway		
LC-MS	AH	(s)-actinidine, 1,2,3,4,6,7,8-heptachlorodibenzofuran, 2,2'-iminodipropan-l-ol, 2(n)-methyl-norsalsolinol, 3-methoxytyramine, 5-methoxytryptophol, acetaminophen, cerulenin, decanoylcarnitine, hippuric acid, indoleacetaldehyde, I-octanoylcarnitine, levofloxacin, imsp01080031, n-(2,4-dimethylphenyl)formamide, n-acetylarylamine, n-acetylornithine, n,n-dimethyl-safingol, nadolol, netilmicin, orsellinic acid, oxybuprocaine, p-cresol sulfate, pantothenic acid, paracetamol sulfate, pyridoxal, pyrocatechol sulfate, sodium tetradecyl sulfate, spisulosine, theophylline, tryptophol	Taurine and hypotaurine metabolism, vitamin B6 metabolism, pantothenate, and coenzyme A biosynthesis	5-methoxytryptophol and cerulenin	94
UHPLC-MS/MS	Vitreous	L-carnitine, propionylcarnitine, theophylline, glycerol tripropanoate, theobromine, gamma glutamyltyrosine, L-kynurenine, pivaloylcarnitine, 1-(beta-D-R-ribofuranosyl)-1,4-dihydronicotinamide, isobutyry-L-carnitine, N-acetylornithine, L-2,3-dihydrodipicolinate,N-acetylhistidine, monoethylglycinexylidide, 5-hydroxyindoleacetic acid, aspartame, L.acetylcarnitine, dehydroneotenone, 4-guanidinobutanoic acid, uric acid, 16-methylheptadecanoic acid, indoxyl sulfate, estradiol	Tryptophan metabolic pathway, uric acid metabolic pathway, oxidative stress and inflammation	——	95

## Abbreviations

AH: aqueous humor
AKT: serine-threonine kinase
AL: axial length
APOA1: apolipoprotein A1
cGMP: cyclic guanosine monophosphate
DBP: vitamin D-binding protein
ECM: extracellular matrix
EGF: epidermal growth factor
FDM: form-deprivation myopia
GDF: growth differentiation factor
G-CSF: granulocyte colony stimulating factor
HM: high myopia
HGF: hepatocyte growth factor
HIF-1: hypoxia-inducible factor-1
IGF: insulin-like growth factor
IGFBP: IGF-binding protein
IL: interleukin
IL-1ra: IL-1 receptor antagonist
IFN-γ: interferon-γ
IP-10: interferon-γ-induced protein 10
JAK: janus tyrosine kinase
MAPK: mitogen-activated protein kinase
mCNV: myopic choroidal neovascularization
MCP-1: monocyte chemoattractant protein-1
MIP-1α: macrophage inflammatory protein-1α
MMP: matrix metalloproteinase
NGS: next-generation sequencing
NO: nitric oxide
PDGF: platelet-derived growth factor
PEDF: pigment epithelium-derived factor
PI3K: phosphatidyl-inositol 3-kinas
PM: pathologic myopia
PKG: protein kinase G
ROS: reactive oxygen species
sICAM-1: soluble intercellular adhesion molecule-1
svCAM-1: soluble vascular cell adhesion molecule-1
STAT: signal transducer and activator of transcription
T-AOC: total antioxidant capacity
TGF-β: transforming growth factor-β
TIMP: tissue inhibitors of metalloproteinase
TNF: tumor necrosis factor
VEGF: vascular endothelial growth factor
